# Supplementary figures and images for: Mitogenomics and Evolutionary History of Rodent Whipworms (Trichuris spp.) Originating from Three Biogeographic Regions
Source: Life (Basel). 2021 Jun 9;11(6):540. doi: 10.3390/life11060540 (PMC8228637; doi:10.3390/life11060540)

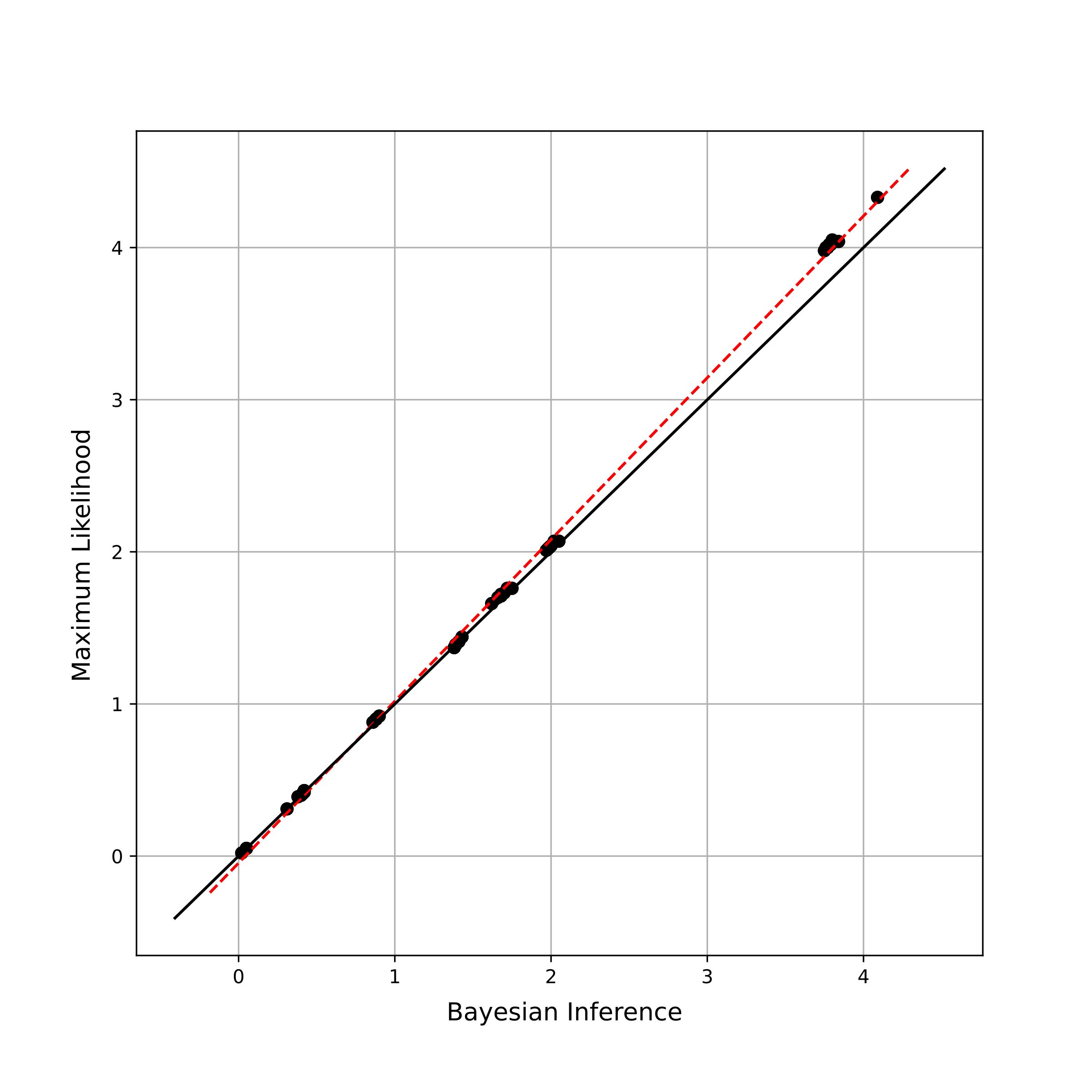

Supplement: Supplementary file 1 [file life-11-00540-s001.zip › Figure_S2.png]

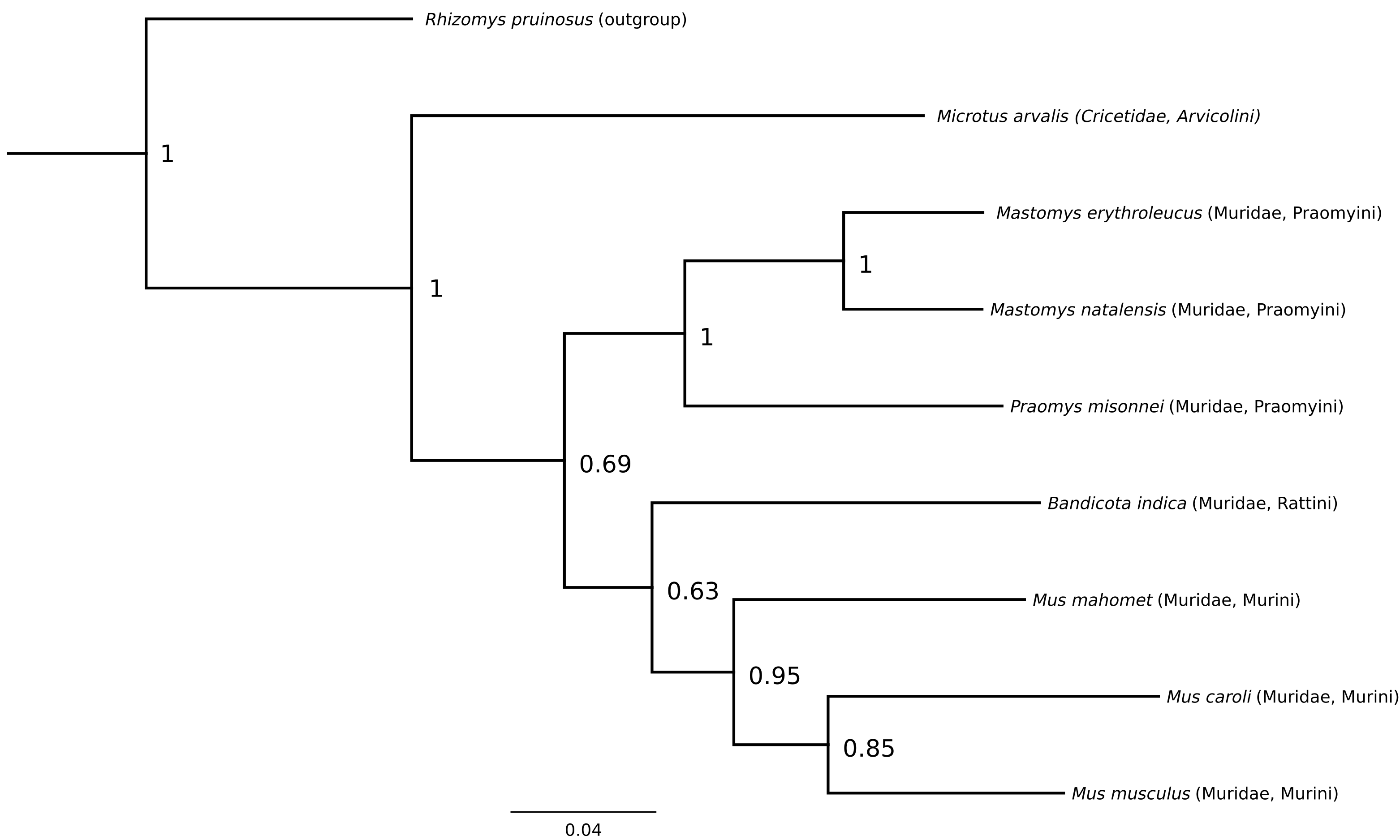

Supplement: Supplementary file 1 [file life-11-00540-s001.zip › Figure_S3.png]
